# Supplementary material for: Long arcuate fascicle in wild and captive chimpanzees as a potential structural precursor of the language network
Source: Nat Commun. 2025 May 15;16:4485. doi: 10.1038/s41467-025-59254-8 (PMC12081605; doi:10.1038/s41467-025-59254-8)
Supplement: Supplementary file 1 — Supplementary Information [file 41467_2025_59254_MOESM1_ESM.pdf]

Supplementary information for:
Long arcuate fascicle in wild and captive chimpanzees as a possible structural precursor of the language network

Y. Becker, C. Eichner, M. Paquette, C. Bock, C. Girard-Buttoz, C. Jäger, T. Gräßle, T. Deschner, EBC Consortium, P. Gunz, R. M. Wittig, C. Crockford, A. D. Friederici, A. Anwander

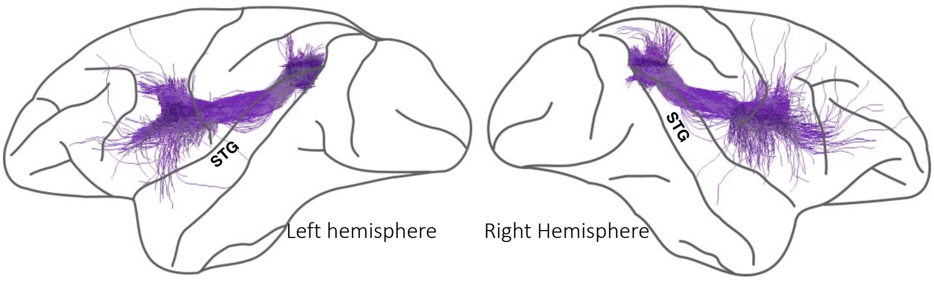

Supp. Fig. S1. Probabilistic tractography of the arcuate fascicle in a macaque monkey.
The figure shows the reconstruction of the arcuate fascicle in high resolution post-mortem diffusion MRI data. Note that the arcuate fascicle connects only to the posterior STG and no other parts of temporal lobe. Adapted from Balezeau et al., Nat. Neurosci. 2020.

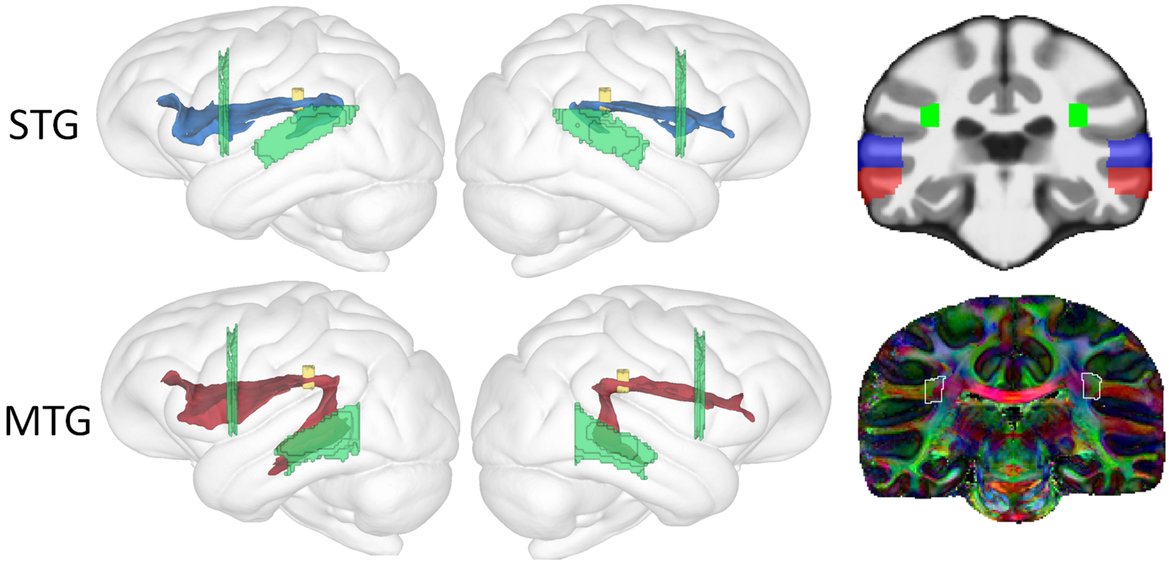

Supp. Fig. S2. Seed and waypoint masks for probabilistic tractography of the arcuate fascicle in chimpanzees.
The figure shows the seed ROIs (yellow) in the core of the arcuate fascicle and the temporal and frontal waypoint masks (green) for AF-STG and AF-MTG tractography together with the

group averaged tract in blue and red in the chimpanzee template. Only the masks in the temporal lobe differ between the tracking of the two components of the AF. Right panel show the seed ROI on a coronal slice on the template (top) and on the colour FA map of a single subject (bottom). All masks are provided as supplementary files.

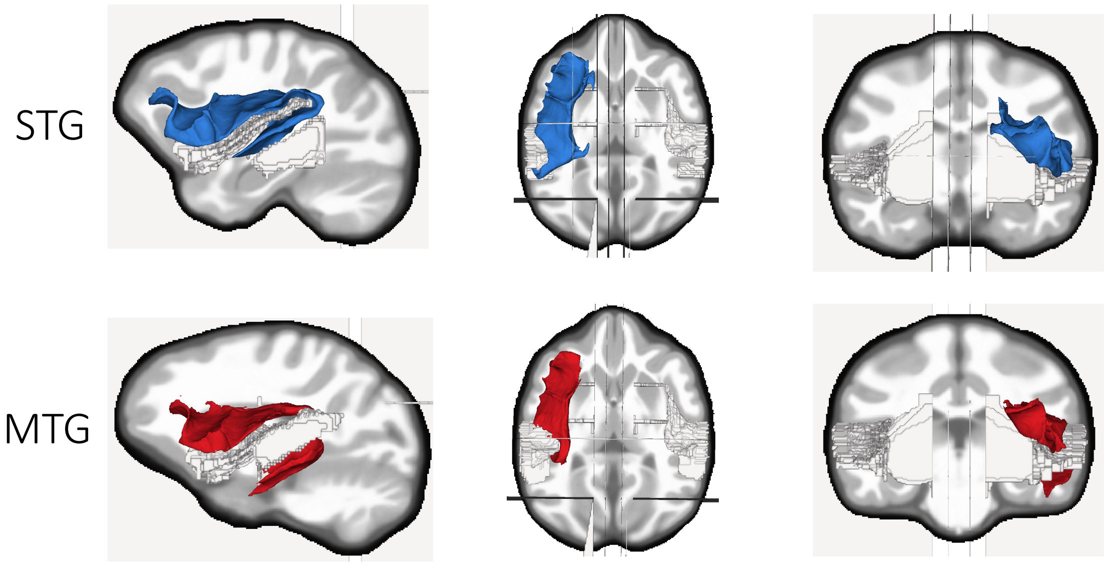

Supp. Fig S3. Exclusion masks for probabilistic tractography of the arcuate fascicle in Chimpanzees.
The figure shows the exclusion masks (grey) for AF-STG (blue) and AF-MTG (red) connections in the chimpanzee template. The exclusion masks between the AF-STG and the AF-MTG tractography differ only in the temporal lobe. The AF-STG tracking used the MTG mask (See also Supp. Fig 3) as exclusion mask, and the AF-MTG tracking used the STG mask as exclusion mask. All masks are provided as supplementary files.

Comparisons between probabilistic and systematic deterministic tractography

An additional systematic and observer-independent deterministic tractography was implemented to validate the AF-STG and AF-MTG connections and replicate the quantification of connectivity strength. The deterministic tractography was based on the same local fibre crossing and tracking model as in the virtual dissection, but used the frontal, parietal and temporal ROIs from the probabilistic tractography to select the connecting streamlines.

Methods: For deterministic tractography, we used the same local ODF fibre crossing and tracking model as for virtual dissection. We seeded 100 streamlines per voxel in a conservatively selected large white matter region containing all potential branches of the arcuate fascicle. Tractography was computed in a full white matter mask using the following

parameters: relative ODF threshold: 0.1, step size: 0.5 mm, maximum streamline length: 100 mm, angular threshold: 85°, tracking in both directions. Streamlines for the AF-STG and AF-MTG pathways were selected using the same frontal, parietal and temporal ROIs as in the probabilistic tractography model. To account for potential partial volume effects in streamline selection, all selection ROIs were dilated by 0.5 mm.

Results: A comparison between probabilistic and deterministic tractography shows quasi-identical connectivity patterns. Overall, the streamline count from the more conservative deterministic tractography unsurprisingly resulted in lower relative connectivity values than the probabilistic tractography (see Supp. Fig. S4). This is also partly related to linear scaling effects due to a lower number of seeded streamlines used in this model. Supp. Fig. S5 shows a comparison of all individual probabilistic and deterministic tractography results.

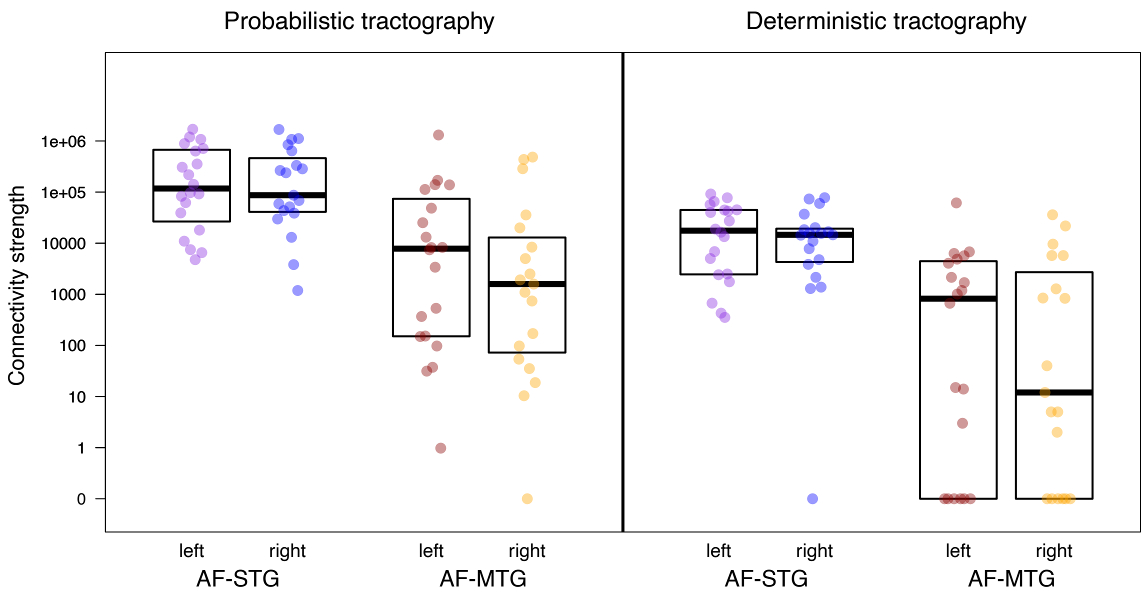

Supp. Fig. S4: Comparison between deterministic and probabilistic tractography results in chimpanzees.

Probabilistic (left) and deterministic (right) tractography results show the same relative connectivity pattern of stronger AF-STG than AF-MTG connectivity (right hemisphere N=19; left hemisphere N=20). The streamline count in deterministic tractography shows lower connectivity values than probabilistic tractography due to differences in the number of initially seeded streamlines and the generally more conservative method. The dots show the individual connectivity values. The thick horizontal line represents the median and the box the 25% and 75% quartiles.

Individual tractography results

Deterministic Probabilistic Deterministic Probabilistic

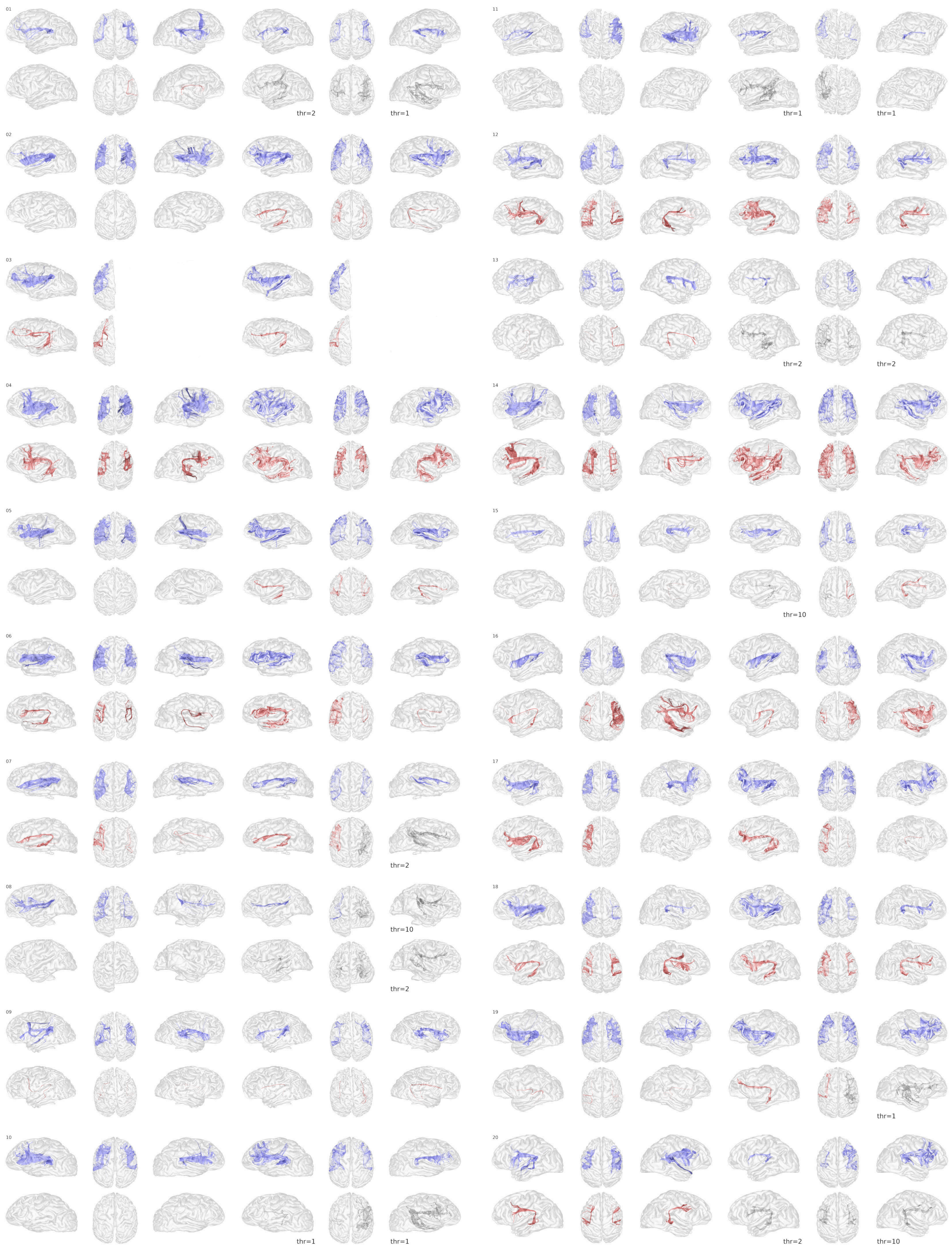

Supp. Fig. S5: Individual results of deterministic and probabilistic tractography for AF-STG and AF-MTG in left and right hemispheres.

Note the quasi-identical morphology for each individual. The probabilistic tractography results were classified into weak connections for individuals with a probabilistic connectivity below 1000 (displayed as a grey isosurface showing the tract volume with an individual threshold), and strong connections for all individuals with a connectivity values of more than 1000 (displayed as a blue (AF-STG) or red (AF-MTG) isosurface showing the tract volume with more than 100 streamlines per voxel). The AF-MTG connections in two individuals were not robustly detected (#10, #11 with total probabilistic connectivity strength below 50) and were excluded from the lateralisation analysis.

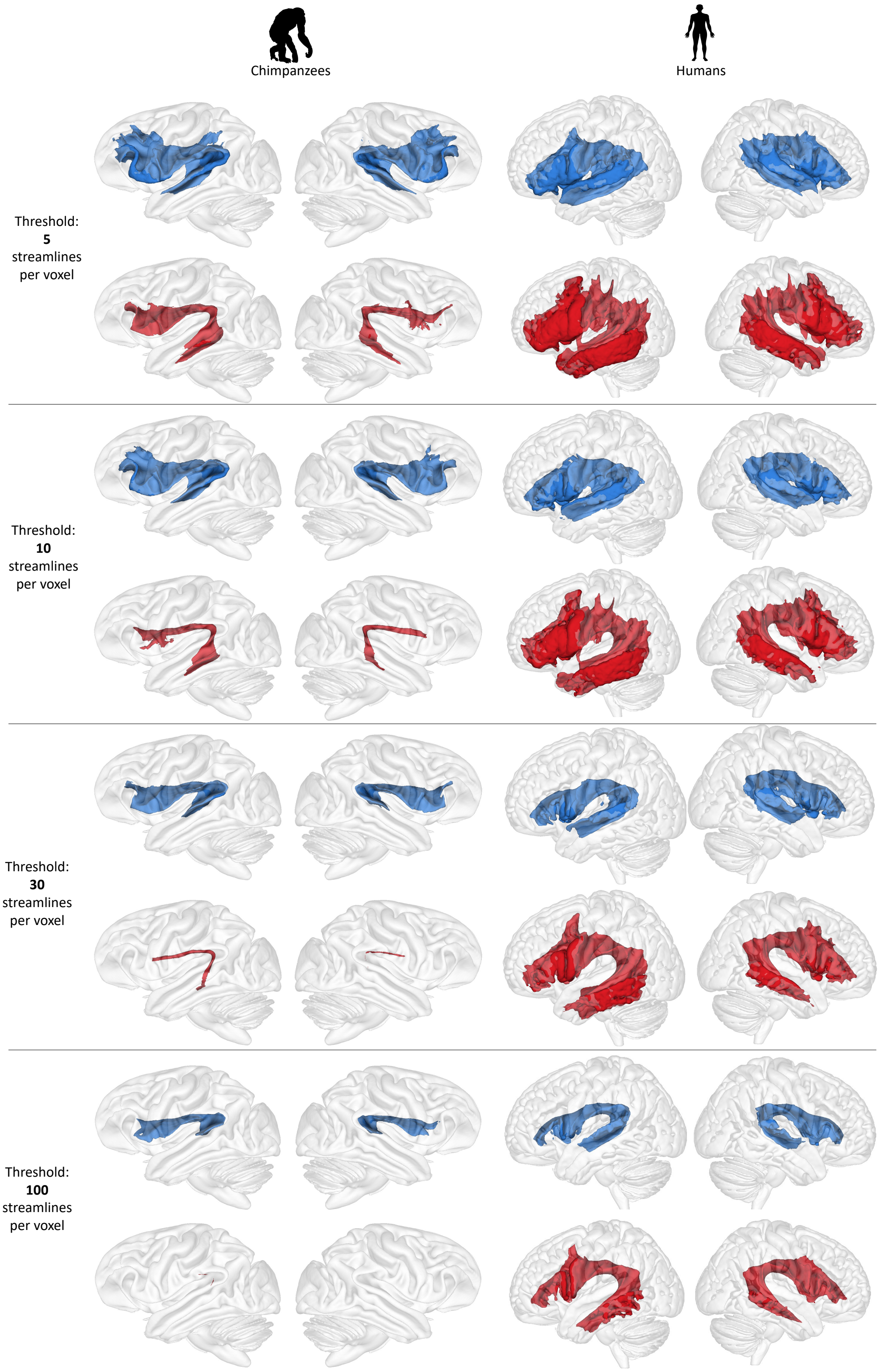

Supp. Fig. S6. Group-level averaged tractography results displayed with different thresholds for chimpanzees and humans.

Probabilistic tractography for AF-STG (blue) and AF-MTG (red) connections of the arcuate fascicle displayed on the chimpanzee and human template. AF-STG and AF-MTG connections are shown as isosurfaces displayed for 5 (top) to 100 (bottom) probabilistic streamlines per

voxel. The group mean tractography results are generated in three steps: 1. Intensity scaling of the maps with the number of streamlines per voxel to an average seed size of 1000 voxels. 2. Logarithmic scaling of the maps to reduce the dynamic range and approximate a Gaussian intensity distribution. 3. Spatial normalisation to the group template and averaging across subjects. The final maps are rescaled to represent the average number of streamlines per voxel. The 3D connectivity maps without thresholding are available as supplementary file.

Human tractography

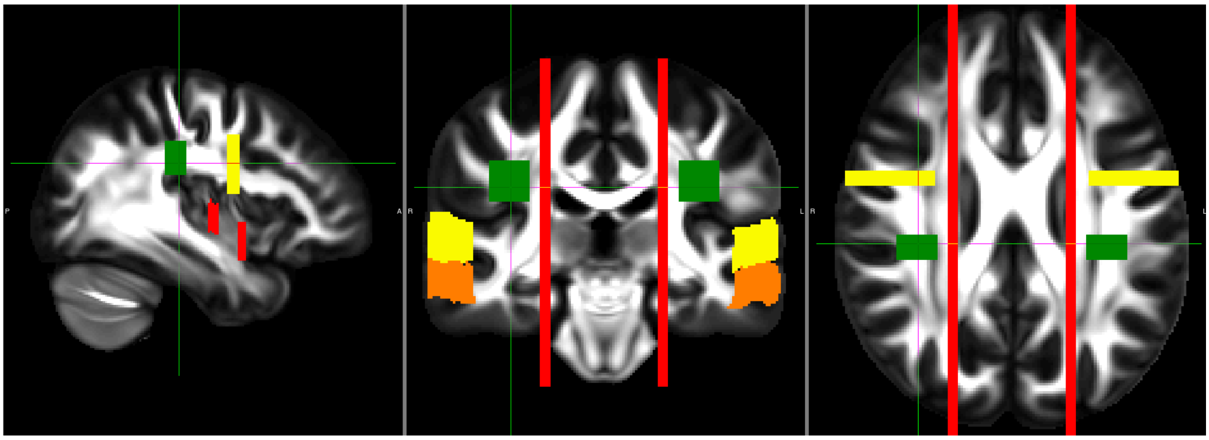

Supp. Fig. S7. ROIs for human comparative tractography.
The images show the seed ROI (green), the frontal and temporal tractography ROIs (yellow and orange) and the exclusion ROIs (red) for probabilistic tractography superimposed on the human FSL_HCP1065_FA template in MNI space.

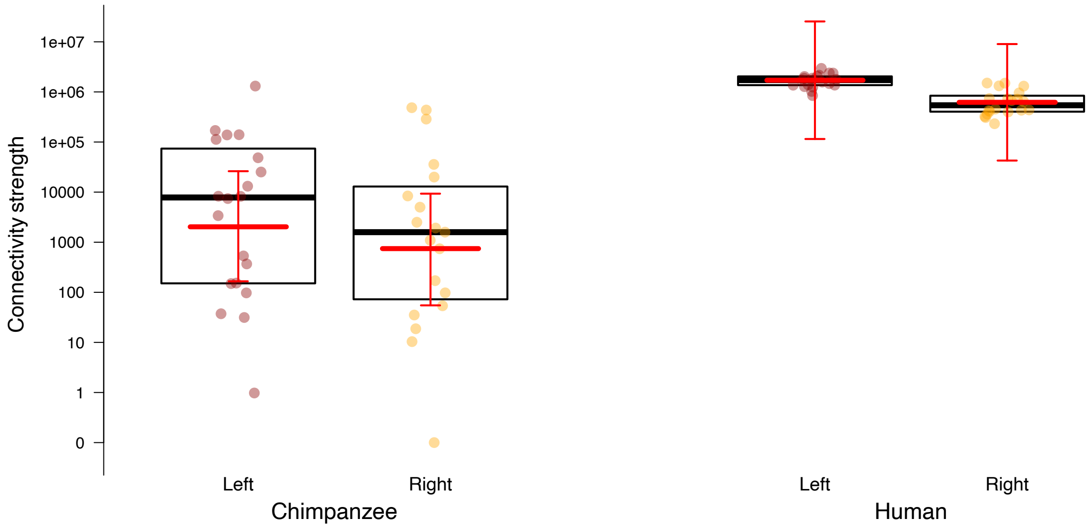

Supp. Fig. S8. AF-MTG lateralisation of human and chimpanzee.

The plot shows the connectivity strength of the left and right AF-MTG connection for chimpanzees (left panel) (left hemisphere: N=20; right hemisphere: N=19) and humans (right panel) (each hemisphere: N=20). The dots show the individual connectivity values. The thick horizontal lines represent the median values and the box the 25% and 75% quartiles. The red horizontal lines represent the model means estimates with the 95% CI.

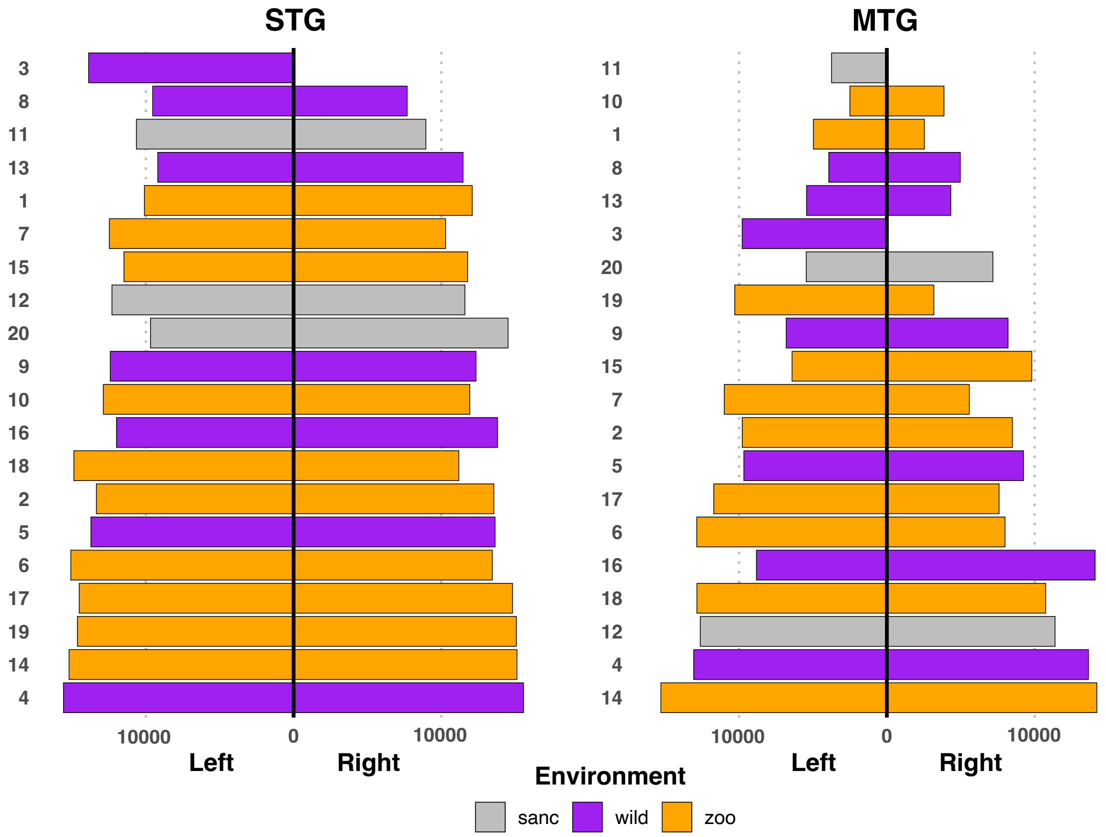

Supp. Fig. S9. AF-STG and AF-MTG connectivity strength by living environments.
Individual AF-STG (left) and AF-MTG (right) connectivity values ordered by strength (sum of right and left hemispheres). Wild individuals (purple, N=7), zoo-housed individuals (orange, N=10), sanctuary-housed individuals (grey, N=3). The graphs show no influence of the living environment on the individual connection strength.
